# Physicochemical Characterization and Biological Properties of Polysaccharides from *Alpiniae oxyphyllae* Fructus

**DOI:** 10.3390/polym16121705

**Published:** 2024-06-14

**Authors:** Risi Wang, Xinmei Ruan, Jun Chen, Lizhen Deng, Wei Zhou, Xixiang Shuai, Ruihong Liang, Taotao Dai

**Affiliations:** 1 School of Food Science and Engineering, Jiangxi Agricultural University, Nanchang 330045, China; wangrisi@jxau.edu.cn; 2 Key Laboratory of Tropical Fruit Biology of Ministry of Agriculture and Rural Affairs, South Subtropical Crop Research Institute, China Academy of Tropical Agricultural Sciences, Zhanjiang 524091, China; 3 State Key Laboratory of Food Science and Technology, Nanchang University, Nanchang 330047, China; 4 Key Laboratory of Tropical Crop Products Processing of Ministry of Agriculture and Rural Affairs, Agricultural Products Processing Research Institute, Chinese Academy of Tropical Agricultural Sciences, Zhanjiang 524001, China

**Keywords:** polysaccharides, *Alpiniae oxyphyllae* fructus, extraction methods, physicochemical properties, antioxidant activities, α-amylase inhibition

## Abstract

Polysaccharides (AOPs) were extracted from *Alpiniae oxyphyllae* fructus using three distinct methods: hot water (AOP-HW), hydrochloric acid (AOP-AC), and NaOH/NaBH4 (AOP-AL). This study systematically investigated and compared the physicochemical properties, structural characteristics, antioxidant activities, and α-amylase inhibitory activities of the extracted polysaccharides. Among the three AOPs, AOP-AC exhibited the highest yield (13.76%) and neutral sugar content (80.57%), but had the lowest molecular weight (121.28 kDa). Conversely, AOP-HW had the lowest yield (4.54%) but the highest molecular weight (385.42 kDa). AOP-AL was predominantly composed of arabinose (28.42 mol%), galacturonic acid (17.61 mol%), and galactose (17.09 mol%), while glucose was the major sugar in both AOP-HW (52.31 mol%) and AOP-AC (94.77 mol%). Functionally, AOP-AL demonstrated superior scavenging activities against DPPH, hydroxyl, and ABTS radicals, whereas AOP-AC exhibited the strongest inhibitory effect on α-amylase. These findings indicate that the extraction solvent significantly influences the physicochemical and biological properties of AOPs, thus guiding the selection of appropriate extraction methods for specific applications. The results of this study have broad implications for industries seeking natural polysaccharides with antioxidant and enzymatic inhibitory properties.

## 1. Introduction

*Alpiniae oxyphyllae* Miq., belonging to the species of *Zingiberaceae*, is an herbal medicine distributed between the Yangtze River and the Nan Mountains in China. The dried mature fruit of *Alpiniae oxyphyllae* (*A. oxyphyllae*) Miq. was considered to have the potential to warm the kidney, reduce the urine, and store essential substances in traditional Chinese medicine theory [1,2,3]. In China, *A. oxyphyllae* fructus has been used on a large scale as a Chinese medicine material. *A. oxyphyllae* fructus mainly contains polysaccharide, essential oil, terpene, flavonoid, and heptane derivatives [4]. Modern pharmacological studies have proved that *A. oxyphyllae* fructus polysaccharide (AOP) shows a good efficacy in urinary incontinence, anti-neuro inflammatory activity, antiviral activity, and immunoregulation [1,5,6,7,8]. Yang et al. characterized the structure of *A. oxyphyllae* fructus polysaccharide and found that it could significantly increase the phagocytic activity of RAW264.7 macrophages [9]. Generally, the bioactivity of polysaccharides depends on the chemical composition and structural features, which varies with a range of factors, especially the extraction solvent employed [10,11,12].

Extraction conditions, including temperature, pH, and the type and concentration of the extracting solvents, can directly influence the structure of the obtained polysaccharide. Solvents have significant effects on the biological activities and physicochemical features of polysaccharides [13,14]. Water, acid, and alkali are common extracting agents, selected based on the nature of the polysaccharide. Hot water extraction is widely utilized for polysaccharide extraction because it is simple, low-cost, safe, and environment-friendly [15,16]. Acidic and alkali solutions are also usually used to extract polysaccharides. It has been reported that acidic solvents are effective in cleaving glycoside linkages within polysaccharides, leading to increased yields of small-molecule polysaccharides [17,18]. Moreover, several relevant studies have found that alkali-extracted polysaccharides possess higher bioactivities, such as anti-inflammatory and antioxidant properties [19,20]. Zhang, Zhu, Zhang, Yang, Ni, Zhang, Liu, and Zhang [21] extracted polysaccharides from green tea leaf using different extraction solvents and found that alkali-extracted polysaccharide showed a higher viscosity and better gelling properties than hot-water- and acid-extracted polysaccharides. Therefore, it is necessary to compare the physicochemical and biological properties of polysaccharides with different solvents. Thus far, the research about polysaccharides from *A. oxyphyllae* fructus has all involved water extraction [1,5,6]. The effect of different extraction solvents on the physicochemical and biological properties of polysaccharides of *A. oxyphyllae* fructus is still unclear.

In summary, the objective of this study was to evaluate the effects of different solvents (hot water, hydrochloric acid, and NaOH/NaBH_4_) on the physicochemical (chemical composition, molecular weight (Mw), morphology, and crystal structure) and biological (antioxidant and α-amylase inhibitory ability) properties of polysaccharides extracted from *A. oxyphyllae* fructus. This work might provide valuable information for the utilization of AOPs in the food and dietary supplements industries.

## 2. Result and Discussion

### 2.1. Extraction Yields and Physicochemical Properties of AOPs

#### 2.1.1. Extraction Yields

In general, the yield of polysaccharides from different sources depends on the extraction parameters and raw materials. As shown in Table 1, the yield of AOPs varies with the extraction solvent. AOP-AC had the highest yield (13.76%), followed by AOP-AL (8.88%) and AOP-HW (4.54%). The higher yields of AOP-AC and AOP-AL may result from the disruption of the cell wall structure under acidic and alkali conditions, which accelerated the release of soluble polysaccharides into the extract [22]. During the extraction process, hot water only dissolves long-chain soluble polysaccharides, resulting in the lowest extraction yield of AOP-HW. Zhang, Zhu, Zhang, Yang, Ni, Zhang, Liu, and Zhang [21] also found that the yield of green tea leaf polysaccharides extracted by alkali and acid solution was higher than that by hydrothermal extraction. In the acidic extract, the glycosidic bond between biopolymers was disrupted by H+, and the soluble molecules quickly dissolved towards the extracellular space under the electrostatic stress, resulting in a significant increase in the production of polysaccharides [23].

#### 2.1.2. Chemical Composition

The uronic acid, neutral sugar, and protein contents of the three AOPs are shown in Table 1. The neutral sugar contents of AOP-AC and AOP-HW were 80.57% and 69.62%, respectively, significantly higher than that of AOP-AL (28.28%). This result may be interpreted as the fact that the glycosidic bonds in the AOPs are more sensitive, and the acidic environment promotes its hydrolysis [22]. The uronic acid contents of AOP-AL, AOP-HW, and AOP-AC were 63.13%, 19.51%, and 13.15%, which indicated that the three AOPs were all anionic polysaccharides. This result also indicated that AOP-AL was an acidic polysaccharide. This finding may be due to the fact that alkaline conditions were conducive to the release of uronic acid, thus increasing the content of uronic acid in the AOP-AL. All AOPs were contaminated with protein, but the protein content was low (1.12–3.37%). It was possible that the obtained polysaccharides were deproteinized by the Sevag reagent and the remaining protein was closely connected to the framework of polysaccharides.

**Figure 1:**
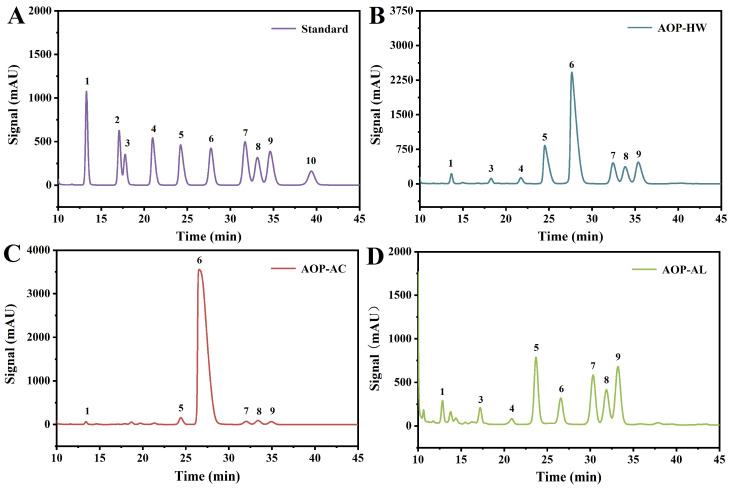
HPLC spectra of monosaccharide composition. (**A**) Monosaccharide standards, (**B**) AOP-HW, (**C**) AOP-AC, and (**D**) AOP-AL. Peaks: (1) mannose, (2) ribose, (3) rhamnose, (4) glucuronic acid, (5) galacturonic acid, (6) glucose, (7) galactose, (8) xylose, (9) arabinose, and (10) fucose. The samples AOP-HW, AOP-AC, and AOP-AL represent polysaccharides extracted from *A. oxyphyllae* fructus using hot water, hydrochloric acid, and NaOH/NaBH_4_, respectively.

The monosaccharide composition results of the three AOPs were presented in Table 2 and Figure 1. There were certain differences in the relative abundance of each polysaccharide. Glucose was the predominant sugar found in AOP-HW (52.31 mol%) and AOP-AC (94.77 mol%), which was in line with Han’s study (96.8 mol%) [1]. Glucose was found to account for an exclusively high proportion of the hot-water-extracted polysaccharides from the same *Zingiberaceae* family, such as *Kaempferia galanga* L (34.10 mol%) and *Zingiber* officinale (96.00 mol%) [24,25]. However, the main monosaccharides of AOP-AL were determined as arabinose (28.42 mol%), galactose (17.64 mol%), and galacturonic acid (17.09 mol%), indicating that AOP-AL was likely to be hemicellulose [26]. And the monosaccharides of the AOP-HW sample mainly included arabinose, glucose, and xylose, which was speculated to be araboxylan [27]. As the glucose content of AOP-AC exceeds 90%, it was presumed to be glucan. This finding was aligned with a previous study which showed that the polysaccharides extracted from *Alpiniae oxyphyllae* fructus were composed of glucose alone [9]. Their exact structure has yet to be further resolved by means such as nuclear magnetic resonance. The above results indicated that extraction solvents affected the chemical composition of the AOPs.

#### 2.1.3. Mw of AOPs

**Figure 2:**
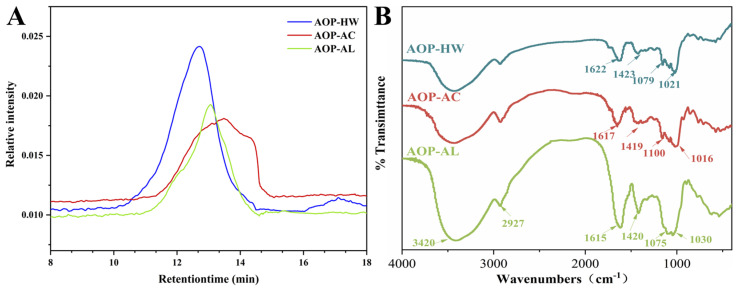
Molecular weight curves (**A**) and FT-IR spectra (**B**) of three polysaccharides extracted from *A. oxyphyllae* fructus with different extraction methods. The samples AOP-HW, AOP-AC, and AOP-AL represent polysaccharides extracted from *A. oxyphyllae* fructus using hot water, hydrochloric acid, and NaOH/NaBH_4_, respectively.

The Mw of AOPs detected by high-performance size exclusion chromatography is displayed in Table 1 and Figure 2A. The molecular weight profiles of AOP-HW, AOP-AC, and AOP-AL all contained a single RI peak, and their Mw values were 385.42, 121.28, and 232.40 kDa, respectively. The lower average Mw of AOP-AL and AOP-AC indicated that polysaccharides were degraded during the extraction process due to harsh extraction conditions. A similar trend was found in the previous study; the Mw decreased with the increase in H^+^/OH^-^ concentration, and polysaccharides gradually degraded from large molecules to small-size polymers [28]. Both AOP-HW and AOP-AL exhibited symmetrical peaks, while AOP-AC had a broad molecular mass distribution. The relatively wide Mw distribution of AOP-AC might be due to the cleavage of glycosidic bonds and intramolecular hydrogen bonds of AOP-AC to form a variety of glucose polymers during the extraction process, inferring this polysaccharide may have a highly branched and heterogeneous structure [19].

#### 2.1.4. FT-IR Spectroscopy Analysis

The FT-IR spectra of AOP-HW, AOP-AC, and AOP-AL are depicted in Figure 2B. The absorption peak between 3100 and 3700 cm^−1^ corresponds to the O−H stretching vibration caused by inter- and intramolecular hydrogen bonds [29]. The band at 2800–3000 cm^−1^ is associated with the stretching vibration of C−H in unconstrained carbohydrates [5]. The strong absorption peak at 1615–1653 cm^−1^ is accounted for by the C=O asymmetric stretching vibration of the carbonyl group, while the weak band at 1420 cm^−1^ reflects the C−O stretching vibration of the carbonyl group [30]. The peak at 1090 cm^−1^ indicates the existence of a pyranose ring in the main chain [25]. The characteristic absorption at 1020 cm^−1^ might represent an α-1,6 linkage [30,31]. The three AOPs by FT-IR analysis demonstrated the typical absorption peaks of polysaccharides. A small shoulder at ca. 1730 cm^–1^ is visible for AOP-HW which is accounted for by the C=O stretching vibration of the methyl esterified carboxylic group. However, the C=O stretching vibration of the methyl esterified carboxylic group is not shown in the FT-IR profile of AOP-AC and AOP-AL, which indicates that de-esterification possibly occurs during acidic or alkali extraction.

### 2.2. Morphological Properties

**Figure 3:**
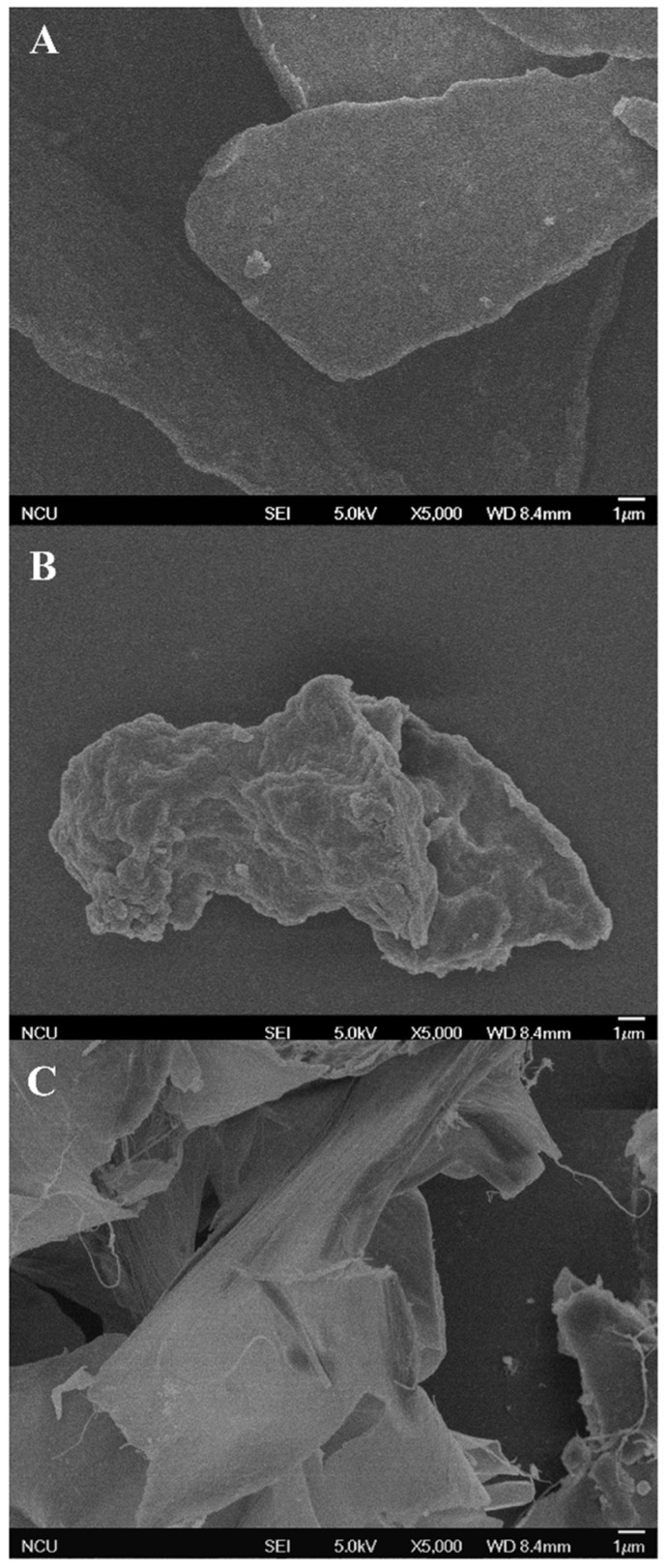
Scanning electron micrographs of (**A**) AOP-HW, (**B**) AOP-AC, and (**C**) AOP-AL at 5000-fold magnification. The samples AOP-HW, AOP-AC, and AOP-AL represent polysaccharides extracted from *A. oxyphyllae* fructus using hot water, hydrochloric acid, and NaOH/NaBH_4_, respectively.

The SEM images provide the surface morphological properties of the AOPs. The morphologies of the three AOPs presented significant differences in shapes and sizes (Figure 3). The microstructure of AOP-HW appeared an irregular and slightly rough layer with uneven dimensions (Figure 3A), which was similar to that reported by Shi, Zhong, Zhang, and Yan [5]. AOP-AL possessed many thick and smooth slice patches, and some filamentous connections were found on the surface (Figure 3C). However, AOP-AC exhibited a block appearance with uneven dimensions, which was composed of many corrosive hill-like and irregular pores (Figure 3B). Based on the principle of hydrochloric acid extraction, it could be inferred that the polysaccharide molecules were effectively decomposed into small segments by H^+^ [32], which was in line with the abovementioned Mw distribution and yield of AOP-AC.

### 2.3. XRD Analysis

**Figure 4:**
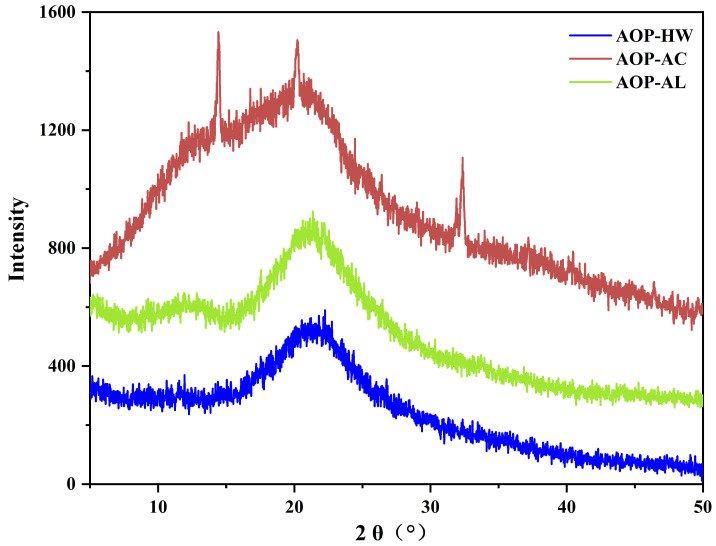
X-ray diffraction patterns of three polysaccharides extracted from *A. oxyphyllae* fructus with different extraction methods. The samples AOP-HW, AOP-AC, and AOP-AL represent polysaccharides extracted from *A. oxyphyllae* fructus using hot water, hydrochloric acid, and NaOH/NaBH_4_, respectively.

XRD was applied to examine the crystalline structure of the three AOPs. Generally, sharp and narrow peaks reflect crystalline structures, while broad peaks indicate amorphous structures. As shown in Figure 4, AOP-HW and AOP-AL displayed no particularly obvious diffraction peaks between 5° and 50°, indicating that these two polysaccharides were amorphous polymers. Most studies have reported that water-extracted polysaccharides from plants are amorphous structures without obvious crystallization peaks [33,34]. Meanwhile, AOP-AL was likely to be derived from hemicellulose, because it contained a high content of arabinose, galactose, and galacturonic acid. Soluble hemicellulose is a group of complex plant polysaccharides arranged in disorder [35], thus AOP-AL was an amorphous structure. However, AOP-AC was a crystalline structure with major diffraction peaks at 14.45°, 26.25°, and 32.38°. These diffraction peaks might be related to the complex and distinctive molecular organization of cellulose, which corresponds to the elevated abundance of glucose in the monosaccharide composition of AOP-AC. These observations suggest that the extraction process was closely related to the structure of the polysaccharides.

### 2.4. In vitro Antioxidant Activity

#### 2.4.1. DPPH Radical Scavenging Activity

**Figure 5:**
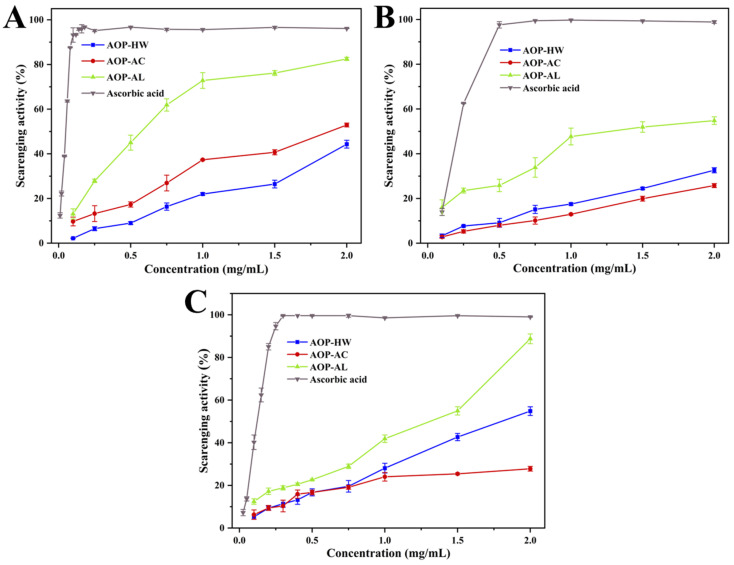
The antioxidant activities of three polysaccharides extracted from *A. oxyphyllae* fructus with different extraction methods. (**A**) DPPH radical scavenging activity, (**B**) hydroxyl radical scavenging activity, (**C**) ABTS radical scavenging activity. The samples AOP-HW, AOP-AC, and AOP-AL represent polysaccharides extracted from *A. oxyphyllae* fructus using hot water, hydrochloric acid, and NaOH/NaBH_4_, respectively.

The DPPH radical, a kind of stable free radical source, has been widely used in the evaluation of natural active compounds [36,37]. Figure 5A shows the scavenging activity of the three AOPs against DPPH radicals. The DPPH radical scavenging activities of AOP-HW, AOP-AC, and AOP-AL increased with concentration, achieving peak values of 44.33%, 52.90%, and 82.51% at a concentration of 2.0 mg/mL, respectively. AOP-AL displayed a remarkably higher scavenging activity against DPPH radicals (IC_50_ of 0.56 mg/mL) than AOP-HW (IC_50_ of 2.16 mg/mL) and AOP-AC (IC_50_ of 1.74 mg/mL) (Table 3). These findings suggested that AOP-AL is a potential scavenger of DPPH radicals.

#### 2.4.2. Hydroxyl Radical Scavenging Activity

The hydroxyl radical is a physiological free radical with the greatest lethality on biological components [38]. As shown in Figure 5B, three AOPs exhibited a moderate scavenging ability of the hydroxyl radical. At the concentration of 2.0 mg/mL, the hydroxyl radical scavenging rate of AOP-AL was 1.84 and 2.33 times that of AOP-HW and AOP-AC, respectively. AOP-AL was a considerably effective hydroxyl radical scavenger with an IC_50_ value of 1.07 mg/mL, which was stronger than the alkali-extracted Laminaria japonica polysaccharide (IC_50_ of 1.75 mg/mL) [18] and Zingiber officinale Roscoe stem and leaf polysaccharide (IC_50_ of 2.426 mg/mL) [39]. These results revealed that AOP-AL exhibited an excellent scavenging activity on hydroxyl radicals, which was consistent with the results of the DPPH radical scavenging activity.

#### 2.4.3. ABTS Radical Scavenging Activity

The ABTS radical, a well-known nitrogen-centered synthetic radical, has been widely used to evaluate the antioxidant capacity of polysaccharides [22,40]. At the concentration of 2.0 mg/mL, the scavenging rates of AOP-HW, AOP-AC, and AOP-AL were 54.84%, 27.81%, and 88.76%, respectively (as illustrated in Figure 5C). Among the three AOPs, AOP-AL showed the lowest IC_50_ value (1.40 mg/mL) on the ABTS radical scavenging ability, followed by AOP-HW (2.10 mg/mL) and AOP-AC (3.63 mg/mL), which were stronger than those of the results reported by Amamou, Lazreg, Hafsa, Majdoub, Rihouey, Le Cerf, and Achour [41].

Generally speaking, the types of antioxidants can be summarized as hydrogen atom transfer and single electron transfer [42]. The antioxidant potential of polysaccharides is closely related to their structure characteristics, including chemical composition, type of glycosidic linkage, and monosaccharide composition and conformation [33]. It was suggested that polysaccharides containing a high uronic acid content were effective antioxidants, because their electrophilic groups could promote the release of hydrogen from the O−H bond [43]. In this study, AOP-AL exhibited the best effective antioxidant activity, which was probably due to it having the highest content of uronic acid. Moreover, AOP-HW demonstrated a superior scavenging activity compared to AOP-AC in both the ABTS and hydroxyl radical models. A possible explanation is that AOP-HW had a higher galactose content than AOP-AC. Previous studies have reported that the antioxidant activities of polysaccharides may be explained by their abundant galactose content [44,45].

### 2.5. Inhibitory Effects on α-Amylase Activity

The rise of postprandial blood glucose is related to the hydrolysis of starch by α-amylase. Therefore, the α-amylase inhibition assay can be considered as a rapid and simple strategy to evaluate the in vitro hypoglycemic activity [46].

**Figure 6:**
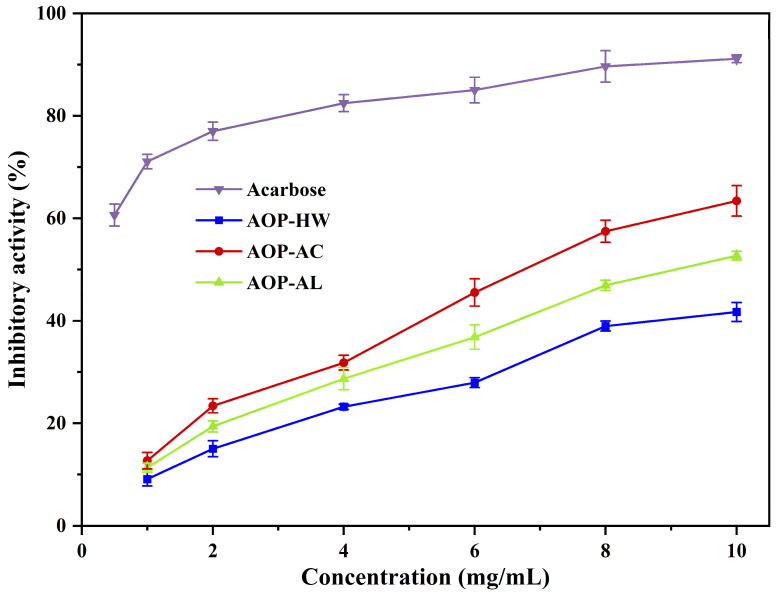
Inhibitory effects of three polysaccharides extracted from *A. oxyphyllae* fructus with different extraction methods on the α-amylase activity. The samples AOP-HW, AOP-AC, and AOP-AL represent polysaccharides extracted from *A. oxyphyllae* fructus using hot water, hydrochloric acid, and NaOH/NaBH_4_, respectively.

As depicted in Figure 6, the inhibitory effects of the three AOPs on α-amylase activity intensified with higher concentrations. At the concentration of 10 mg/mL, the inhibitory abilities of AOP-AC, AOP-AL, and AOP-HW on α-amylase were 63.41%, 52.71%, and 41.73%, respectively, which were higher than those of polysaccharides extracted from *Trifolium pretense L*. and comfrey root (20–35%) [37,47]. AOP-AC possessed the strongest inhibitory effect on α-amylase activity among the three AOPs. Amamou, Lazreg, Hafsa, Majdoub, Rihouey, Le Cerf, and Achour [41] reported that anti-hyperglycemic activity might be related to the high amounts of glucose in the polysaccharide structure. Meanwhile, the high content of uronic acid could also inhibit the enzyme, because the amino acid residues of α-amylase easily interact with free carboxyl groups [13], which verified that AOP-AL had a stronger α-amylase inhibitory activity than AOP-HW despite the low glucose content.

## 3. Materials and Methods

### 3.1. Materials and Reagents

*Alpiniae oxyphyllae* fructus was collected from Guangxi Renjitang Pharmaceutical Co. (Guangxi, China). In total, 10 monosaccharide standards including galacturonic acid, xylose, mannose, arabinose, rhamnose, ribose, glucose, galactose, glucuronic acid, and fucose were provided by Yuanye Biotechnology Co., LTD. (Shanghai, China). Acarbose, α-amylase (3700 U/g), 1,1-diphenyl-2-picrylhydrazyl (DPPH), and dextran standards were purchased from Sigma-Aldrich Co. (Shanghai, China). All other chemicals were obtained from Solarbio Science & Technology Co., LTD. (Beijing, China).

### 3.2. Polysaccharide Extraction and Isolation

*Alpiniae oxyphyllae* fructus were separately extracted using an acid solution, hot water, and alkaline solution. The specific extraction conditions were chosen based on a combination of referenced literature [48] and our preliminary experimental findings. In brief, 100.0 g of *A. oxyphyllae* fructus powder was degreased with *n*-hexane (1:5 w/v) for 6 h, and then extracted with 3 L of distilled water at 90 °C for 2 h, 3 L of hydrochloric acid (pH 1.5) at 90 °C for 2 h, and 3 L of 0.1 mol/L NaOH/20 mmol/L NaBH_4_ solution at 25 °C for 2 h, respectively. After centrifugation (5000 rpm, 5 min), the supernatant was neutralized and concentrated with a rotary evaporator. The crude polysaccharide was isolated by adding four volumes of ethanol, allowing the mixture to stand for 12 h, and then centrifuging it at 5000 rpm for 20 min. The polysaccharide solution was then deproteinized with Sevag reagent and dialyzed (8–14 kDa) for 3 days. The three dehydrated polysaccharides were obtained by freeze-drying (FD5 series, SIM International Group, LA, USA), namely, AOP-HW, AOP-AC, and AOP-AL.

The extraction yield of crude *A. oxyphyllae* fructus polysaccharide was calculated using Formula (1):

(1) Yield(%)=[(C×V×d)/m]×100

where *C* represents concentration of polysaccharides (mg/mL), *V* represents volume of extraction solution (mL), *d* denotes dilution ratio, and *m* represents weight of *A. oxyphyllae* fructus powders (g).

### 3.3. Determination of Chemical Composition

The contents of neutral sugar, uronic acid, and protein were determined according to the phenol–sulfuric method [49], carbazole–sulfuric acid method (galacturonic acid as the standard) [49], and spectrophotometric method (bovine serum albumin as the standard) [50], respectively.

### 3.4. Monosaccharide Composition Analysis

The monosaccharide composition of AOPs was analyzed using an HPLC system (1260 series, Agilent Technologies, USA) equipped with a diode array detector and a Thermo ODS-2 column (4.6 × 250 mm, 5 μm) [17]. In brief, a 2.0 mg polysaccharide sample was hydrolyzed in trifluoroacetic acid (2 mol/L) for 2 h (120 °C). After removing the trifluoroacetic acid by nitrogen flushing, 0.5 mL of 1-phenyl-3-methyl-5-pyrazolone methanol solution (0.5 mol/L) and 0.5 mL of NaOH solution (0.3 mol/L) were added to the sample. The mixture was heated at 70 °C for 30 min and neutralized with hydrochloric acid. Subsequently, 0.5 mL of chloroform was added, and the extraction was repeated three times. The aqueous layers were combined for HPLC analysis. The chromatographic conditions were as follows: UV detection wavelength (254 nm), mobile phase = phosphate buffer (0.1 mol/L, pH 7.0) and acetonitrile (82:18, v/v), column temperature = 25 °C, flow rate = 1.0 mL/min, injection volume = 20 μL [1].

### 3.5. Determination of Mw

The Mw of AOPs was analyzed by high-performance size exclusion chromatography (Agilent 1260, Agilent Technologies, Santa Clara, CA, USA) equipped with an RI detector and a PL aquagel-OH mixed-M column (7.5 × 300 mm, 8 μm) [51]. The polysaccharide was dissolved in ultrapure water containing 0.02% NaN_3_ and filtered through a 0.45 μm syringe filter. The chromatographic analysis was conducted under the following conditions: column temperature set at 30 °C, flow rate maintained at 0.5 mL/min, injection volume of 20 μL. In addition, calibration curves were established using dextran standards with known molecular weights (Mw: 4.32, 12.6, 60.6, 110, and 496 kDa).

### 3.6. FT-IR Spectroscopy

The functional groups of AOPs were analyzed by FT-IR spectroscopy [52]. The sample was fully ground with dried KBr powder and then tabled into a transparent sheet. A Nicolet 5700 spectrometer (Thermo Nicolet Co., Waltham, MA, USA) was used to obtain spectra in the wavelength range of 4000–400 cm^−1^.

### 3.7. Scanning Electron Microscopy Analysis (SEM)

The dried polysaccharide powder was pasted on a conductive pile and sputtered with a platinum layer [53]. The SEM image was observed using a scanning electron microscope (JEOL Ltd., Akishima, Tokyo, Japan). The detailed measurement parameters were set to the acceleration voltage of 5 kV and the magnification of 5000-fold.

### 3.8. X-ray Diffraction Analysis (XRD)

The crystal structure of AOPs were analyzed using an X-ray diffractometer (D8 Advance, Bruker, Germany) [54]. The measurement was carried out between 5° and 50° (2θ). The counting time and step size were set to 1 s/step and 0.02°, respectively.

### 3.9. Antioxidant Qctivities

#### 3.9.1. DPPH Radical Scavenging Activity

The antioxidant activities of AOPs were assessed using the methodology outlined in a previous literature [33]. Specifically, a mixture containing 1 mL of 0.2 mmol/L DPPH solution (dissolved in 50% ethanol) and 1 mL of polysaccharide solution (at concentrations ranging from 0 to 2 mg/mL) was observed at 25 °C in darkness for 30 min. Subsequently, the absorbance was measured at 517 nm using a TU-1810 spectrophotometer, with distilled water serving as the blank control. The results were computed based on Formula (2):

(2) $${\mathrm{Scavenging}\;\mathrm{activity}\;(\%)=(1-A}_{1}{/A}_{0})\;\times \;100\%$$

where *A*_0_ represents the absorbance of 1 mL distilled water plus 1 mL DPPH solution, and *A*_1_ shows the absorbance of 1 mL polysaccharide solution to be measured plus 1 mL DPPH solution.

#### 3.9.2. Hydroxyl Radical Scavenging Activity

The hydroxyl radical scavenging activities were analyzed according to the method reported by Wei and Zhang [38]. In summary, an aliquot of 1 mL containing 2 mmol/L FeSO_4_ and another aliquot of 1 mL with 6 mmol/L ethanol–salicylic acid were introduced to 1 mL of a polysaccharide solution. Subsequently, this amalgamation was combined with 1 mL of 6 mmol/L H_2_O_2_ while being continuously agitated. Following this, the resulting mixture was subjected to incubation for a duration of 30 minutes at a temperature of 37 °C. The optical density was then measured at a wavelength of 510 nm using a TU-1810 spectrophotometer. The hydroxyl radical scavenging activity was calculated according to Formula (3):

(3) $$\mathrm{Scavenging}\;\mathrm{activity}\;(\%)=\;{[1-(A}_{2}{-A}_{1}{)/A}_{0}]\;\times \;100\%$$

where *A*_0_ represents the absorbance of the reaction system containing 1 mL FeSO_4_, 1 mL ethanol–salicylic acid solution, 1 mL H_2_O, and 1 mL H_2_O_2_; *A*_1_ shows the absorbance of the reaction system containing 1 mL FeSO_4_, 1 mL ethanol–salicylic acid solution, 1 mL polysaccharide solution, and 1 mL H_2_O; and *A*_2_ denotes the absorbance of the reaction system containing 1 mL FeSO_4_, 1 mL ethanol–salicylic acid solution, 1 mL polysaccharide solution, and 1 mL H_2_O_2_.

#### 3.9.3. ABTS Radical Scavenging Activity

The ABTS radical scavenging activity was measured in accordance with the procedures outlined in the literature [31] with certain adjustments. Initially, the ABTS stock solution was formulated following the documented protocol and subsequently diluted using phosphate buffer (pH 7.0) to achieve an absorbance of 0.700 ± 0.030 at 734 nm. Subsequently, a mixture of 30 μL of polysaccharide solution and 970 μL of ABTS diluent was prepared in a 48-well microplate. Following a 6 min incubation period in darkness, the absorbance was measured at 734 nm using a microplate reader. The ABTS radical scavenging activity was estimated by Formula (4):

(4) $$\mathrm{Scavenging}\;\mathrm{activity}\;(\%)\;{=[1-(A}_{0}{-A}_{1}{)/A}_{0}]\;\times \;100\%\;$$

where *A*_0_ denotes the absorbance value of the blank control, while *A*_1_ indicates the absorbance value of the sample.

### 3.10. α-Amylase Inhibitory Activity

The α-amylase inhibitory activity was performed as reported by Duan, Shang, Chen, Li, and Wu [42]. Briefly, α-amylase solution (100 μL, 0.5 mg/mL) and phosphate buffer (100 μL, 0.2 mol/L, pH 6.9) were mixed with 100 μL of polysaccharides solution at 37 °C for 10 min. Then, a soluble starch solution (200 μL, 1.0%) was added and incubated for 10 min at 37 °C. The reaction was suspended by adding dinitrosalicylic acid reagent (200 μL) and heating in boiling water for 10 min. Finally, the absorbance was observed at 540 nm by a TU-1810 spectrophotometer. The α-amylase inhibitory activity was calculated according to Formula (5):

(5) $$\;\alpha \text{-}\mathrm{amylase}\;\mathrm{inhibitory}\;\mathrm{activity}\;(\%)\;{=[1-A}_{0}{-A}_{1}{/A}_{0}]\;\times \;100\%$$

where *A*_0_ represents the absorbance of the blank control, and *A*_1_ shows the absorbance of the sample.

### 3.11. Statistical Analysis

All experiments and analyses were replicated thrice, and the results were displayed as the mean ± standard deviation. The origin 2021 (OriginLab, Northampton, MA, USA) and SPSS 26.0 (SPSS Inc., Chicago, IL, USA) software were used to conduct draw the graph and one-way ANOVA, respectively. The *p* < 0.05 represents a significance difference between samples.

## 4. Conclusions

This study demonstrated the successful extraction of polysaccharides (AOPs) from *Alpiniae oxyphyllae* fructus using hot water, hydrochloric acid, and NaOH/NaBH4. The choice of extraction solvent significantly influenced the yield, molecular weight, surface morphology, monosaccharide composition, and crystal structure of the AOPs. Specifically, AOP-HW and AOP-AC were rich in glucose, while AOP-AL predominantly comprised arabinose. Functionally, AOP-AL exhibited a superior antioxidant activity, making it a potential candidate for health supplements and pharmaceuticals targeting oxidative stress. In contrast, AOP-AC showed the strongest α-amylase inhibitory activity, suggesting its use in managing hyperglycemia and as a functional ingredient in diabetic-friendly foods. These findings provide crucial insights into selecting appropriate extraction methods based on desired functional properties, underscoring the potential for tailoring AOPs’ production to specific industrial applications such as natural antioxidants or enzymatic inhibitors.

## Figures and Tables

**Table 1:** Yield (%), neutral sugar content (%), uronic acid content (%), protein content (%), and molecular weight (kDa) of *A. oxyphyllae* fructus polysaccharides extracted using different solvents.

Sample	Yield (%)	Neutral Sugar (%)	Uronic Acid (%)	Protein (%)	Mw (kDa)
AOP-HW	4.54 ± 0.46 ^c^	69.62 ± 2.11 ^b^	19.51 ± 1.21 ^b^	1.12 ± 0.95 ^b^	385.42 ± 5.64 ^a^
AOP-AC	13.76 ± 0.29 ^a^	80.57 ± 0.72 ^a^	13.15 ± 1.10 ^c^	1.24 ± 0.76 ^b^	121.28 ± 5.18 ^c^
AOP-AL	8.88 ± 0.21 ^b^	28.28 ± 1.14 ^c^	63.13 ± 2.58 ^a^	3.37 ± 1.30 ^a^	232.40 ± 3.93 ^b^

The samples AOP-HW, AOP-AC, and AOP-AL represent polysaccharides extracted from *A. oxyphyllae* fructus using hot water, hydrochloric acid, and NaOH/NaBH_4_, respectively. Different letters in the same column represent significant differences (*p* < 0.05). Mw, molecular weight.

**Table 2:** Monosaccharide ratio of three polysaccharides extracted from *A. oxyphyllae* fructus with different extraction methods (mol%).

Samples	Man	Rha	GlcA	GalA	Glc	Gal	Xyl	Ara
AOP-HW	1.52 ± 0.05 ^b^	2.59 ± 0.03 ^b^	1.65 ± 0.04 ^b^	13.54 ± 0.13 ^b^	52.31 ± 0.44 ^b^	6.86 ± 0.10 ^b^	10.08 ± 0.06 ^a^	10.81 ± 0.16 ^b^
AOP-AC	0.30 ± 0.01 ^c^	N.D.	N.D.	1.42 ± 0.04 ^c^	94.77 ± 0.36 ^a^	0.78 ± 0.06 ^c^	1.74 ± 0.02 ^b^	1.00 ± 0.04 ^c^
AOP-AL	3.45 ± 0.11 ^a^	9.84 ± 0.23 ^a^	3.24 ± 0.02 ^a^	17.09 ± 0.21 ^a^	10.33 ± 0.19 ^c^	17.64 ± 0.14 a	10.00 ± 0.07 ^a^	28.42 ± 0.21 ^a^

Samples AOP-HW, AOP-AC, and AOP-AL represent polysaccharides extracted from *A. oxyphyllae* fructus using hot water, hydrochloric acid, and NaOH/NaBH_4_, respectively. Different letters in the same column represent significant differences (*p* < 0.05). Man, mannose; Rha, rhamnose; GlcA, glucuronic acid; GalA, galacturonic acid; Glc, glucose; Gal, galactose; Xyl, xylose; Ara, arabinose; N.D., not detected.

**Table 3:** Scavenging activity of three polysaccharides extracted from *A. oxyphyllae* fructus with different extraction methods for DPPH, hydroxyl, and ABTS radicals.

Samples	IC_50_ (mg/mL)		
	DPPH Radical	Hydroxyl Radical	ABTS Radical
AOP-HW	2.16 ± 0.06 ^a^	2.29 ± 0.14 ^b^	2.10 ± 0.07 ^b^
AOP-AC	1.74 ± 0.08 ^b^	2.79 ± 0.12 ^a^	3.63 ± 0.10 ^a^
AOP-AL	0.56 ± 0.04 ^c^	1.07 ± 0.06 ^c^	1.40 ± 0.08 ^c^
Ascorbic acid	0.05 ± 0.00 ^d^	0.32 ± 0.02 ^d^	0.12 ± 0.03 ^d^

The samples AOP-HW, AOP-AC, and AOP-AL represent polysaccharides extracted from *A. oxyphyllae* fructus using hot water, hydrochloric acid, and NaOH/NaBH_4_, respectively. Different letters in the same column represent significant differences (*p* < 0.05).

## Data Availability

The original contributions presented in the study are included in the article, further inquiries can be directed to the corresponding authors.
